# Study of a three-dimensional biofilm-electrode reactor (3D-BER) that combined heterotrophic and autotrophic denitrification (HAD) to remove nitrate from water†

**DOI:** 10.1039/d3ra01403g

**Published:** 2023-05-15

**Authors:** Xiangyu Lin, Haoran Yin, Lixin Wang, Yini Chen, Fan Zhao, Yu Pu, Xinhua Tang

**Affiliations:** a School of Civil Engineering and Architecture, Wuhan University of Technology Wuhan 430070 China tangxinhua@whut.edu.cn; b College of Engineering, Pennsylvania State University 201 Old Main, University Park PA 16802-15 USA

## Abstract

A three-dimensional biofilm-electrode reactor (3D-BER) that combined heterotrophic and autotrophic denitrification (HAD) was developed to remove nitrate. The denitrification performance of the 3D-BER was evaluated under different experimental conditions, including current intensities (0–80 mA), COD/N ratios (0.5–5), and hydraulic retention times (2–12 h). The results showed that excessive current limited the nitrate removal efficiency. However, a longer hydraulic retention time was not required to achieve a better denitrification effect in the 3D-BER. Moreover, the nitrate could be effectively reduced over a broad range of COD/Ns (1–2.5), and its removal rate peaked at 89% at *I* = 40 mA, HRT = 8 h, and COD/N = 2. Although the current reduced the diversity of microorganisms in the system, it promoted the growth of dominant species. Nitrification microorganisms were enriched in the reactor, especially *Thauera* and *Hydrogenophaga*, which were crucial to the denitrification process. Thus, the combination of autotrophic denitrification and heterotrophic denitrification was promoted by the 3D-BER system to increase the efficiency of nitrogen removal.

## Abstract 2

The 3D-BER enriched denitrifying microorganisms and improved nitrogen removal efficiency.

## Graphical abstract

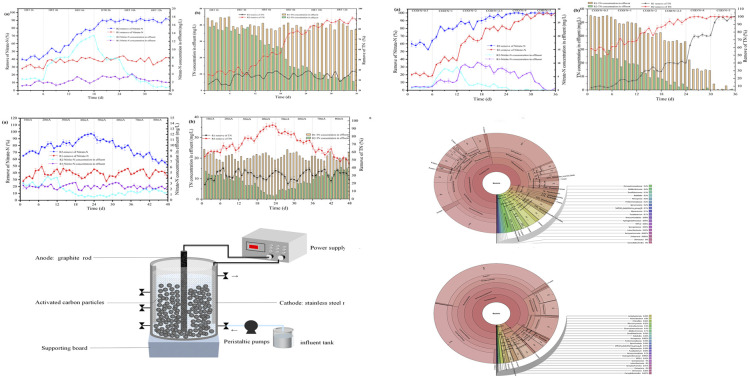

## Introduction

The concentration of nitrate in groundwater has been increasingly severe over most of the world due to the excessive use of chemical fertilizers and the discharge of industrial wastewater in recent years.^1^ Nitrate-derived nitrites and nitrosamines have negatively impacted animal and human health. The nitrites bind to hemoglobin in the blood to create a methemoglobin compound. Its inability to transport oxygen to organs and tissues results in a disease called methemoglobinemia which is characterized by bleeding from the skin and mucous membranes.^2^

The traditional physical and chemical methods for removing nitrate included reverse osmosis, electrodialysis, and ion exchange.^3^ However, these approaches had a high cost and unsatisfactory removal effect. As an alternative, biological denitrification was a more cost-effective method that consumed less energy and fewer chemicals.^4^ As a result, groundwater nitrate removal had benefited considerably from applying biological denitrification technology.^5^ The complete reduction of NO_3_^−^ to N_2_ during denitrification was as follows:^6^ NO_3_^−^ → NO_2_^−^ → N_0_ → N_2_O → N_2_. In order to reduce nitrate, heterotrophic bacteria employed CH_3_OH or CH_3_CH_2_OH as a carbon source.^7^ During autotrophic denitrification using an inorganic carbon source, S or H_2_ were used as electron donors to degrade nitrates. The biofilm-electrode reactor (BER) attracted considerable interest in removing nitrates. This method was more suitable for treating sewage with fewer organic carbons than other methods.^8^ In this method, hydrogen gas (H_2_) produced by electrolytic water acted as an electron donor for denitrifying microorganisms.^9^ The main biological and electrochemical reactions were outlined below:^10,11^

1 2H_2_O + 2e^−^ → H_2_ + 2OH^−^, e^0^ = −0.0 V

2 2NO_3_^−^ + 2H^+^ + 5H_2_ → N_2_ + 6H_2_O

3 2NO_3_^−^ + CH_3_COOH → 4N_2_ + 6H_2_O + 10CO_2_ + 8OH^−^

4 2H_2_O + C → 4H^+^ + CO_2_ + 4e^−^, e^0^ = 0.208 V

5 2H_2_O → O_2_ + 4H^+^ + 4e^−^, e^0^ = 1.2 V

Carbon electrodes were commonly utilized in biofilm-electrode reactors (BERs) due to their favourable properties, such as ease of biofilm formation, high electrical conductivity, and good mechanical strength. Typically, carbon electrodes served as the anode.^12^ Furthermore, the application of an electric field could promote biofilm metabolism and accelerate the breakdown of contaminants. However, experimental running variables like temperature, nitrate concentration, source of carbon, pH, HRT, and current intensity significantly affected BER's denitrification process.^13^ One significant challenge with BER was the lengthy start-up period required for the adaptation of autotrophic bacteria, coupled with the tendency for biofilm to detach from the electrodes.^14^ The availability of inorganic carbon also limited autotrophic denitrification. In particular, 3D-BER, which had recently been developed, was a novel technology. The technology utilized both biofilm and electrochemical processes by incorporating granular activated carbon (GAC) into both the anode and cathode, providing a large surface area for microbial growth and serving as a third bipolar electrode.^15^ These particles would be polarized into multiply charged microelectrodes at the appropriate voltage. Zhao developed a 3D-BER for removing Acid Orange 7 from simulated wastewater.^16^ This result suggested that 3D-BER could be an effective alternative method for dye wastewater before biological pre-treatment. Compared with conventional 2D-electrochemical systems, the efficiency of 3D-electrochemical processes was improved by particle electrodes. Wu used a novel integrated system consisting of 3D-ER and 3D-BERs in series to treat coking wastewater, indicating that this system removed most of COD and TN with low energy consumption.^17^ Huang constructed a 3D-BER for tertiary denitrification, indicating that the approach increased the efficiency of denitrification and eliminated some dissolved organic matter by microbial electro hydrolysis.^18^

In an attempt to further improve denitrification efficiency, the researchers added some soluble organic carbon sources to the BER, enabling the co-existence of autotrophic denitrification (AD) and heterotrophic denitrification (HD). Given the synergistic relationship between HD and AD, the combined use of heterotrophic and autotrophic denitrification (HAD) was more effective at removing nitrate from groundwater. Zhao's intensified biofilm-electrode reactor (IBER) demonstrated that superiority of HAD over AD or HD.^19^ Similarly, Tong's heterotrophic/biofilm-electrode autotrophic denitrification reactor (HAD-BER) achieved efficient nitrate removal across a broad range of current densities and C/N ratios.^20^ The HAD-BER addressed the issue of insufficient carbon sources in traditional hydrogen autotrophic electrode biofilm technology and thus enhanced the denitrification efficiency.

Here, a heterotrophic/three-dimensional biofilm-electrode autotrophic denitrification reactor was developed, in which GAC was filled in the middle as a particle electrode, and a carbon rod and stainless-steel mesh were employed as the anode and cathode, respectively. In order to conduct a comparative experiment and eliminate the influence of other factors, two reactors (R1, R2) with the same structure were designed. Activated carbon particles of equal mass, the same material and size of carbon rod, and stainless-steel mesh were added to R1, identical to those in R2. R1 was a biofilm reactor (BR) without electrical stimulation, supporting only heterotrophic denitrification. R2 was a 3D-BER, with electrical stimulation, enabling electroactivity and the enrichment of different microorganisms in the reactor. This study aimed to (1) explore the effects of hydraulic retention time (HRT), COD/N ratios, and current (*I*) on the nitrogen removal efficiency; (2) assess the proportions of autotrophic and heterotrophic denitrification under different experimental conditions; (3) elucidate the denitrification mechanism in the 3D-BER process under different COD/N ratios; and (4) examine the species and distribution of microorganisms in the reactor.

## Experimental sections

### Experimental equipment and instruments

The essential components of the whole system were the reactor body, peristaltic pump, power supply, and influent tank. The cylindrical plexiglass container had a 5 cm diameter, a 60 cm height, and an effective volume of 600 mL. 410 g GAC was filled in the reactor, occupying about half of the reactor. The cathode was a stainless-steel mesh (Wuhan Industrial Co., Ltd) cylinder set around a particle electrode, which was filled with GAC and had a diameter of 4 mm (Wuhan Liqiang Co., Ltd). The anode was a 5 mm diameter and 60 cm length graphite carbon rod (Sichuan Huamei Co., Ltd), positioned in the centre of the cylindrical reactor. A peristaltic pump (BT100-2J + DG-6, Rongbai Company, China) was used to manage the flow rate of the inlet and outlet. The current flow was recorded using a power supply (FPS-303D, 0–30 V, 0–3 A) in constant current mode, which was capable of maintaining a stable current output during the experiment.

### Experimental start-up and operation

The sludge collected from Longwangzui Wastewater Treatment Plant mainly had *Thauera*, *Simplicispira*, *Pseudomonas*, *Comamonas*, *Acidovorax*, *Geobacter*, *Brachymonas*, *Shewanella*, *Desulfovibrio*. Enriching the denitrifying bacteria was necessary before inoculating the sludge into the reactor. During the enrichment phase, a nutrient solution with a COD/N ratio of 2 was added to the container. Activated carbon particles and anaerobic sludge were mixed (volume ratio of 1 : 1) and enriched under a temperature of 35 °C. The activated carbon particles showed a thick and dark brown biofilm on the surface after about 7–10 days, indicating a mature biofilm was formed. The artificially simulated sewage (30 mg per L NO_3_^−^–N) was delivered to the reactor. The reactor adapted to intermittent water inflow and was not energized during the initial experiment. The HRT was set to 12 h, and the influent pH was maintained at 7. When the nitrate removal rate was stable, which was then energized and changed to continuous water inflow. The current was set to 10 mA, 20 mA, 30 mA, 40 mA and 50 mA over a period of about two weeks, and each current level lasted for 3 days. The reactor was turned on when the nitrate removal rate stabilized at 80%.

### Analytical methods

The water samples were collected from the effluent of each reactor and filtered them through a 0.45 μm membrane for analysis. COD (Cr) was measured by the National Standard of China (GB-11914-89). The concentrations of nitrate, nitrite, and total nitrogen were measured using ultraviolet spectrophotometry (model UV-1800), *N*-(1-naphthyl) ethylenediamine spectrophotometry, and alkaline potassium persulfate digestion ultraviolet spectrophotometry, respectively. Calibration curves and concentration tests were provided in the ESI.† This experiment assumed that the system's synergistic biological and chemical promotion was ignored, and the 3D-BER process was merely seen as a direct superposition of autotrophic and heterotrophic denitrification. The proportions of autotrophic and heterotrophic denitrification were calculated by comparing the removal efficiency of the two reactors.

### Microbial community analysis

**Fig. 1:**
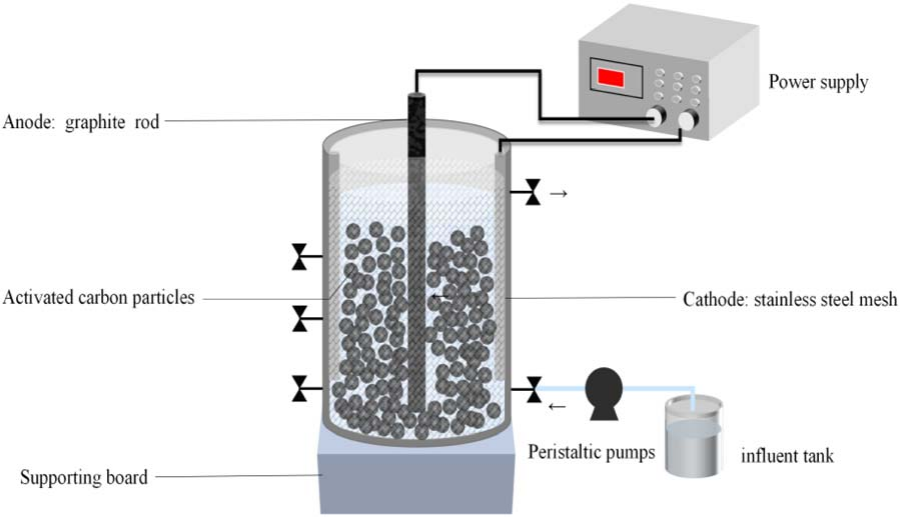
Experimental apparatus.

This experiment measured the microbial community in the reactor's biofilm using 16S ribosomal DNA (rDNA) analysis. It could offer data on a wide range of functional microorganisms.^21^ The GAC sample with biofilm was extracted in about 60 mL from the reactor. The sample was rinsed with deionized water to remove biofilm from the GAC particles. The biofilm mixture was then centrifuged at 4000 rpm for 5 min and stored in a refrigerator at −4 °C until further analysis. The main testing steps for microbial diversity were as follows: DNA extraction, PCR amplification, Construction of PE library, Illumina sequencing, and Bioinformatics analysis (Fig. 1).

## Results and discussion

### Effect of current intensity

Under the conditions of COD/N = 2, HRT = 12 h, pH = 7.5–8.0, and influent NO_3_^−^–N = 30 mg L^−1^, the experiment's current intensity range was from 0 to 80 mA.

**Fig. 2:**
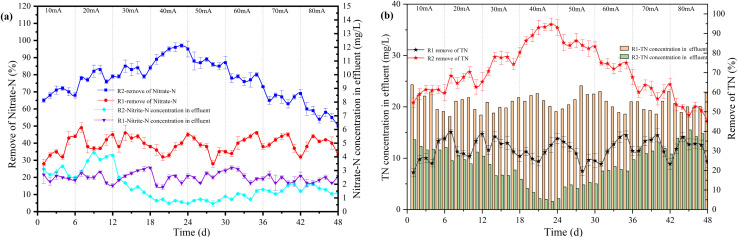
The effect of current intensity on each reactor's denitrification performance.

In R1, which was not energized, only heterotrophic denitrification occurred, resulting in about 38% NO_3_^−^–N removal (Fig. 2). In R2, the removal rates of NO_3_^−^–N and TN increased with the increasing current. When the current intensity increased from 10 mA to 20 mA and 20 mA to 30 mA, the removal rate of NO_3_^−^–N increased from 70% to 79% and 79% to 83%, respectively. A low current intensity (10–40 mA) resulted in a linear relationship between current and denitrification efficiency and the removal rate of NO_3_^−^–N reached a peak of 90% at *I* = 40 mA.^22^ However, “hydrogen suppression” occurred at higher current intensities. The denitrification was inhibited by hydrogen concentrations above a certain level.^23^ Hydrogen would adhere to the surface of microorganisms, which might affect the NO_3_^−^–N mass transfer in the liquid phase. When the current intensity increased from 50 mA to 60 mA and 60 mA to 80 mA, the removal rate of NO_3_^−^–N decreased from 87% to 77% and 77% to 55%, respectively. The TN removal rate was nearly identical to the NO_3_^−^–N and reached a peak of 85% at *I* = 40 mA. The accumulation of NO_2_^−^–N in R1 was about 2.52 mg L^−1^, and there was no apparent change trend. Because of the low influent COD/N ratio, nitrate-reducing bacteria were dominant, and nitrate-reducing bacteria used all organic carbon sources. Part of nitrate was converted to nitrogen, and the other was converted to nitrite. In the R2 reactor, when the current increased from 10 mA to 20 mA, the accumulation of NO_2_^−^–N increased from 2.5 mg L^−1^ to 3.78 mg L^−1^. The denitrification process was still incomplete, resulting in a gradual increase in the concentration of NO_2_^−^–N in the effluent. When the current intensity increased from 30 mA to 40 mA, the accumulation of NO_2_^−^–N reduced from 1.25 mg L^−1^ to 0.8 mg L^−1^. The NO_2_^−^–N could be wholly reduced to nitrogen, resulting in a decrease in the accumulation. The concentration of NO_2_^−^–N in the effluent increased slightly when the current intensity was above than 50 mA.

**Fig. 3:**
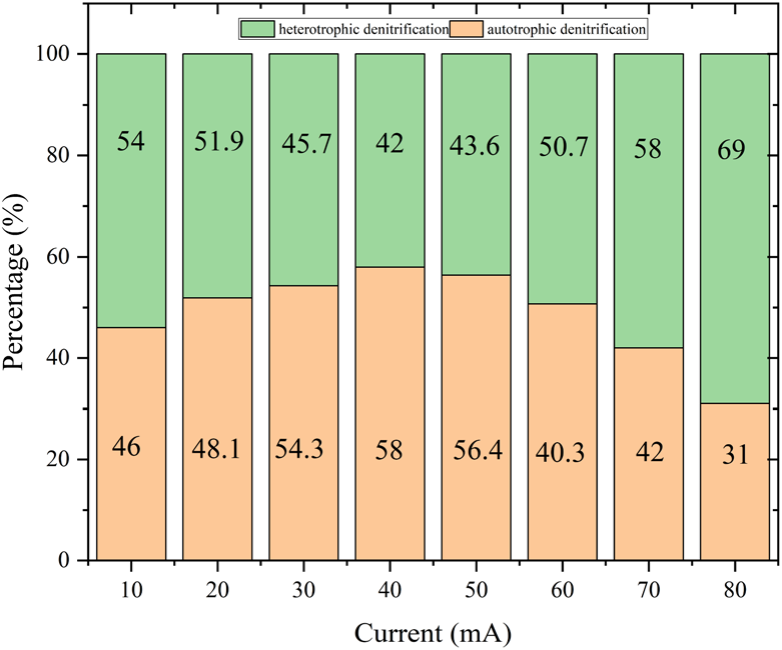
The autotrophic and heterotrophic denitrification proportions in different currents (*I*).

Fig. 3 presented autotrophic and heterotrophic denitrification proportions in different currents (*I*). The autotrophic denitrification proportion increased with increasing current and reached the maximum value of 58% at *I* = 40 mA. As the current intensity increased, so did hydrogen production, electron donor, and denitrification efficiency. The electric current could promote the growth of autotrophic denitrifying microorganisms.^24^ According to research, the current size might affect the growth and activity of microorganisms.^25^ Appropriate electrical stimulation would promote the metabolism of microorganisms and achieve maximum denitrification efficiency.^26^ On the other hand, when the current was 80 mA, the proportion of autotrophic denitrification was only 38%. Excessive electrical current negatively affected microorganisms, destroying cellular components and causing irreversible permeability of cell membranes resulting in leakage of cytoplasmic components.^27^

### Effect of HRT

**Fig. 4:**
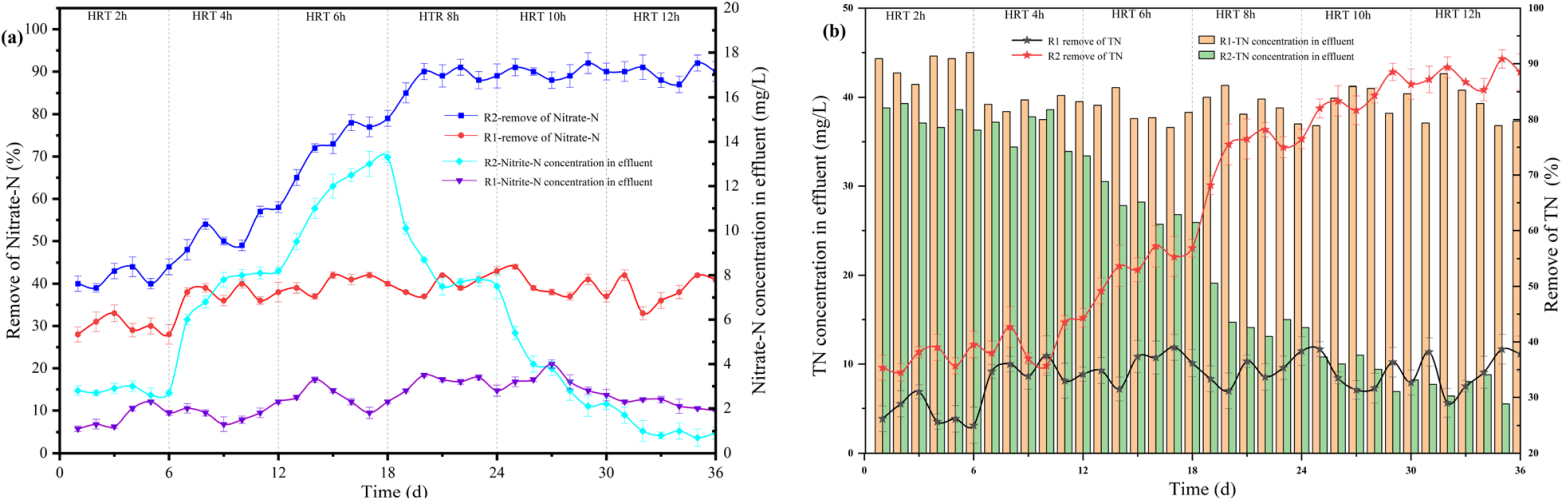
The effect of HRT on each reactor's denitrification performance.

For 3D-BER operation, HRT was a critical parameter. Generally, the longer HRT, the higher the denitrification efficiency. However, if HRT was too long, it was easy to waste resources. If HRT was too short, treatment might not be satisfactory. According to the previous experiments, the reactor operating condition was *I* = 40 mA, pH = 7.5–8.0, COD/N = 2, and the influent NO_3_^−^–N = 60 mg L^−1^. As shown in Fig. 4, the NO_3_^−^–N removal rate was 30% at HRT = 2 h and 38% at HRT = 4 h in R1. With the gradual increase of HRT, the NO_3_^−^–N removal rate maintained at about 38%.

6 0.819 CH_3_COONa + H^+^ + NO_3_^−^ → 0.449 N_2_ + 0.068 C_5_H_7_NO_2_ + 0.301 CO_2_

**Fig. 5:**
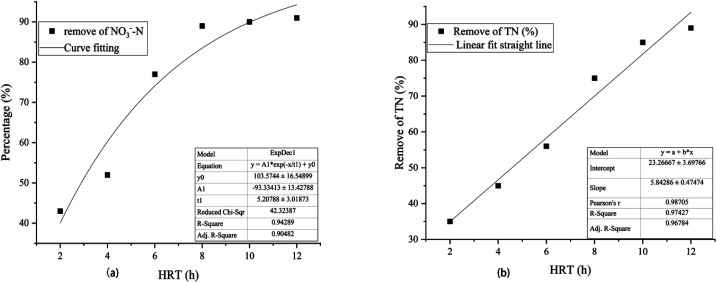
The correlation of HRT with NO_3_^−^–N removal rate and TN removal rate in the 3D-BER.

**Fig. 6:**
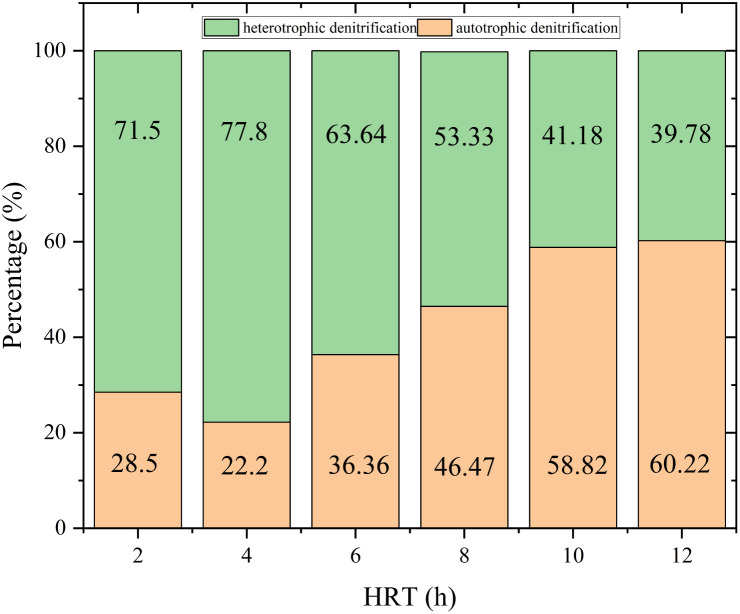
The autotrophic and heterotrophic denitrification proportions in different HRTs.

According to the reaction equation, the theoretical nitrate removal rate was about 50%. The COD concentration in R1 was almost zero. All organic carbon sources (CH_3_COONa) added were used by heterotrophic bacteria for denitrification. When CH_3_COONa was the carbon source, the higher the initial concentration of nitrate, the faster the denitrification rate.^28^ In R2, as shown in Fig. 5, the correlation between HRT and NO_3_^−^–N removal rate was 0.90482. The exponential equation could be expressed: *y* = −93.33413 exp(−*x*/5.20788) + 103.5744, where *y* represented NO_3_^−^–N removal rate and *x* represented HRT. The NO_3_^−^–N removal rate was 43% at HRT = 2 h. The proportion of heterotrophic denitrification was more than 70% (Fig. 6). It could be concluded that the NO_3_^−^–N removal rate of heterotrophic denitrification was usually higher than autotrophic denitrification in the initial stage of the denitrification reaction because the activation energy required by heterotrophic bacteria was lower than autotrophic bacteria.^29^ Firstly, the autotrophic bacteria grew relatively slowly and required more time to degrade nitrogen under a low COD/N ratio. Secondly, in the early stage of the reaction, the electron donor was insufficient, nitrate and nitrite were in a state of competition, and nitrate was in a dominant position When HRT increased from 4 h to 6 h and 6 h to 8 h, the NO_3_^−^–N removal rate increased from 52% to 77% and 77% to 89%, respectively. The proportion of autotrophic denitrification increased from 22.2% to 36.36% and 36.36% to 46.47%, respectively. Nevertheless, HRT was not the bigger, the better. When HRT exceeded 10 h, it was not the decisive factor affecting the denitrification efficiency. The proportion of autotrophic denitrification and NO_3_^−^–N removal rate remained stable. A too-long HRT may lead to the prolonged existence of the endogenous respiration stage of heterotrophic bacteria with low COD/N. It would harm heterotrophic bacteria and worsen the denitrification process.

In the R2 reactor, when HRT increased from 2 h to 4 h and 4 h to 6 h, the accumulation of NO_2_^−^–N increased from 2.81 mg L^−1^ to 7.98 mg L^−1^ and 7.98 mg L^−1^ to 13.33 mg L^−1^, respectively. The accumulation of NO_2_^−^–N gradually decreased with the increase of HRT, reaching 7.93 mg L^−1^ at HRT = 8 h and 0.4 mg L^−1^ at HRT = 12 h. The accumulation of NO_2_^−^–N was higher when HRT was less than 6 h because of the short contact time between microorganisms and nitrate, which resulted in incomplete denitrification and the production of NO_2_^−^–N. Therefore, a longer HRT could promote the denitrification of denitrifying bacteria and reduce the accumulation of NO_2_^−^–N. In addition, the TN removal rate in the R2 reactor increased with the increase of HRT. As shown in Fig. 5, the correlation between HRT and TN removal rate was 0.96784, and the equation could be expressed: *y* = 5.84286*x* + 23.26667, where *y* represented the TN removal rate and *x* represented HRT. When HTR was less than 6 h, the TN removal rate ranged from 40% to 55%. This was mainly because heterotrophic denitrification was the dominant denitrification process in the reactor due to a short HRT, which led to the gradual accumulation of NO_2_^−^–N and affected the TN removal rate.

### Effect of COD/N ratio

Under the conditions of influent NO_3_^−^–N = 60 mg L^−1^, *I* = 40 mA, HRT = 8 h, pH = 7.0–7.5, and temperature = 22–26 °C, a series of influents with different COD/N ratios were prepared (*i.e.*, 0.5, 1, 2, 2.5, 4, 5).

**Fig. 7:**
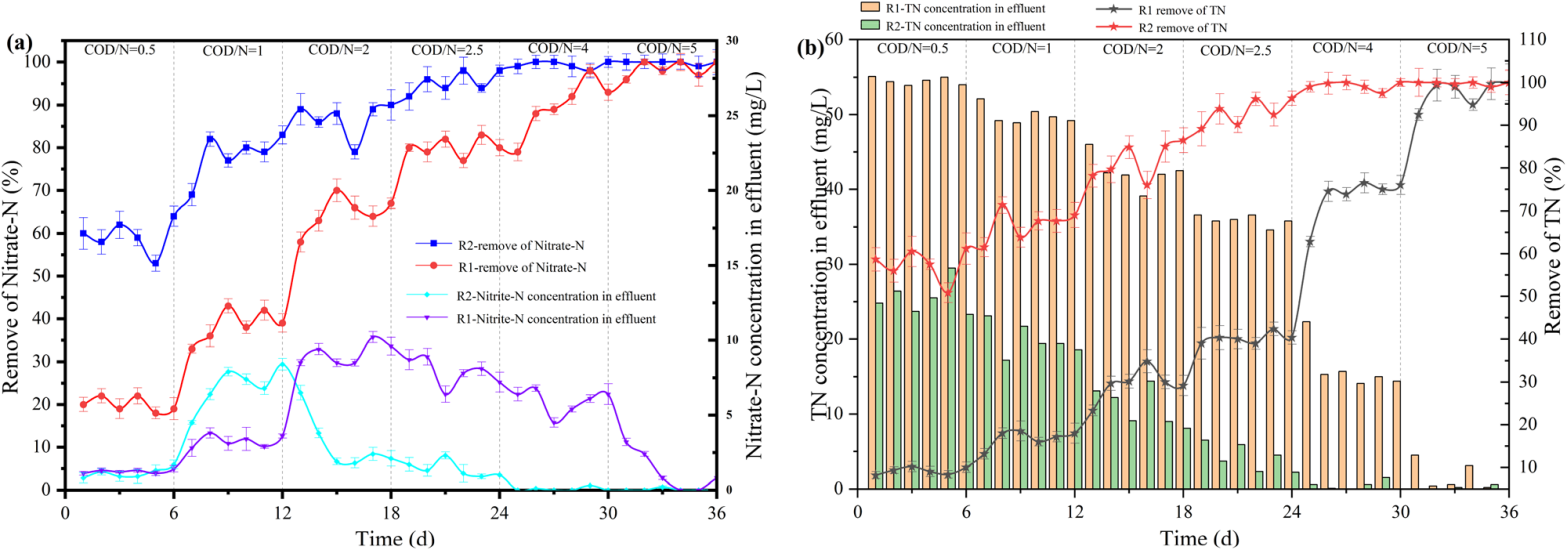
The effect of COD/N ratio on each reactor's denitrification performance.

**Fig. 8:**
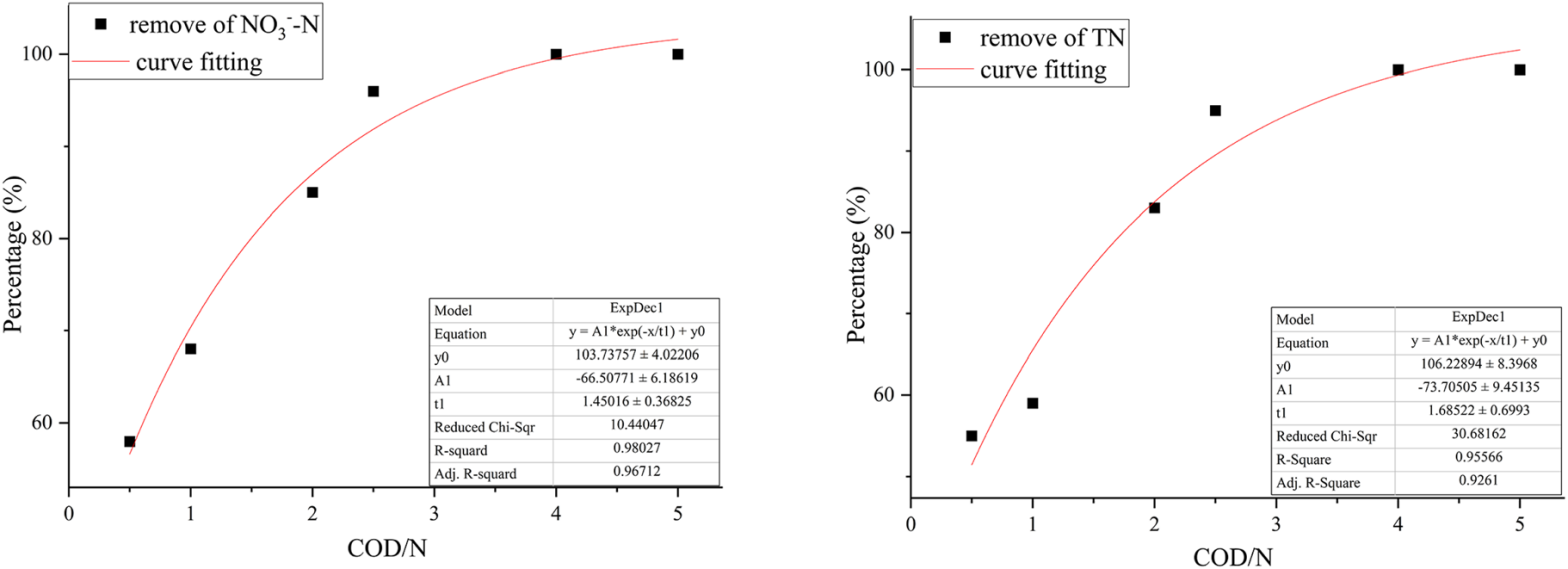
The correlation of COD/N ratio with NO_3_^−^–N removal rate and TN removal rate in the 3D-BER.

The effect of COD/N ratio on the performance of each reactor for denitrification was shown in Fig. 7. In the R1 reactor, when the COD/N ratio increased from 0.5 to 1 and 1 to 2, the removal rate of NO_3_^−^–N increased from 10% to 18% and 35% to 48%, respectively. The nitrate in the water was wholly converted to nitrogen at COD/N = 5. However, the carbon source consumption exceeded the theoretical value, indicating that the heterotrophic denitrification process was not the only denitrification process in the biofilm. Part of the nitrate was removed by assimilatory reduction, which caused biomass growth in the biological reactor.^30^ In the R2 reactor, when the COD/N ratio increased from 0.5 to 1 and 1 to 2, the removal rate of NO_3_^−^–N increased from 62% to 79% and 79% to 86%, respectively. The correlation between the COD/N ratio and NO_3_^−^–N removal rate was 0.96712. The exponential equation could be expressed: *y* = −60.50771 exp(−*x*/1.45016) + 103.73757, where *y* was NO_3_^−^–N removal rate and *x* was COD/N ratio (Fig. 8). The NO_3_^−^–N removal rate was maintained at a high level (>95%) when COD/N > 2.5.

In the R1 reactor, increasing the COD/N ratio from 1 to 2 and from 2 to 2.5 caused the accumulation of NO_2_^−^–N to increase from 1.1 mg L^−1^ to 3.2 mg L^−1^ and from 3.2 mg L^−1^ to 5.8 mg L^−1^, respectively. When the carbon source was insufficient, the first stage of denitrification (NO_3_^−^ → NO_2_^−^) consumed most of the carbon source, leading to the accumulation of NO_2_^−^–N. With the C/N ratio increased, the accumulation of NO_2_^−^–N began to decrease. The accumulation of *v* was 2.1 mg L^−1^ at COD/N = 4 because there was little nitrate in the water, and the carbon source was all used by the nitrite-reducing bacteria. In the R2 reactor, the accumulation of NO_2_^−^–N was 6.93 mg L^−1^ at COD/N = 1. As the COD/N ratio increased from 1 to 2 and from 2 to 2.5, the accumulation of NO_2_^−^–N decreased from 6.93 mg L^−1^ to 3.08 mg L^−1^ and from 3.08 mg L^−1^ to 1.38 mg L^−1^, respectively. The accumulation of NO_2_^−^–N was 0 mg L^−1^ at COD/N > 2.5. The correlation between COD/N and TN removal rate was 0.9261 (Fig. 8). The exponential equation could be expressed: *y* = −73.70505 exp(−*x*/1.68522) + 106.22894, where *y* represented the TN removal rate and *x* represented the COD/N ratio. The TN removal rate of the two reactors differed significantly at the low COD/N (0.5–2.5). A 3D-BER that combined autotrophic and heterotrophic denitrification doubled the TN removal rate.

**Fig. 9:**
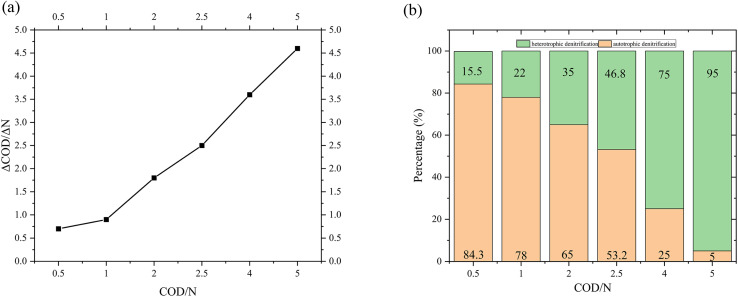
(a) The value of ΔCOD/ΔN under different COD/N ratios in the 3D-BER. (b) The autotrophic and heterotrophic denitrification proportions in different COD/N ratios.

Fig. 9 presented the ΔCOD/ΔN under different COD/N ratios in the 3D-BER. which provide an indicator of denitrification efficiency by representing the number of COD required to remove a unit mass of nitrate nitrogen. As the COD/N ratio increased from 0.5 to 1 and 1 to 2, ΔCOD/ΔN increased from 0.7 to 0.9 and 0.9 to 1.8, respectively. The proportion of autotrophic denitrification (Fig. 9) was 84% at COD/N = 0.5 and 78% at COD/N = 1. It indicated that as the available carbon sources decreased, the activity of heterotrophic microorganisms was limited. Under the action of electric current, heterotrophic denitrifying microorganisms were gradually domesticated into autotrophic microorganisms, leading to their enrichment in the reactor. When the COD/N increased from 2 to 2.5 and 2.5 to 4, ΔCOD/ΔN increased from 1.8 to 2.5 and 2.5 to 3.6, respectively. The proportion of autotrophic denitrification was only 5% at COD/N = 5 and almost all denitrification in the reactor was heterotrophic denitrification. Due to the sufficient carbon sources, heterotrophic microorganisms were enriched in the reactor, improving the efficiency of heterotrophic denitrification. In summary, the 3D-BER primarily conducted autotrophic denitrification at CON/N = 0.5–1, while both autotrophic and heterotrophic denitrifying bacteria played crucial roles at COD/N = 1–2.5. When the COD/N was greater than 2.5, most of the nitrate was removed by heterotrophic denitrification.

### Microbial community

**Fig. 10:**
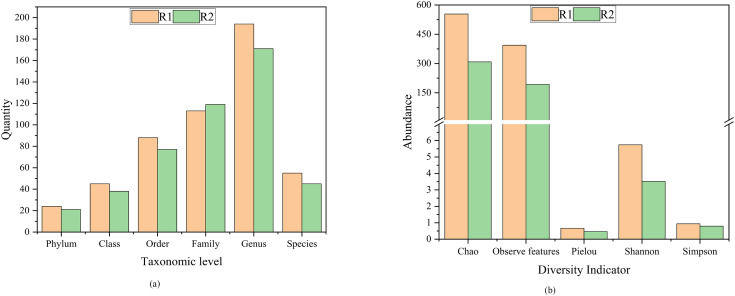
(a) The specific composition of microbial communities at each taxonomic level and (b) microbial diversity indexes.

**Fig. 11:**
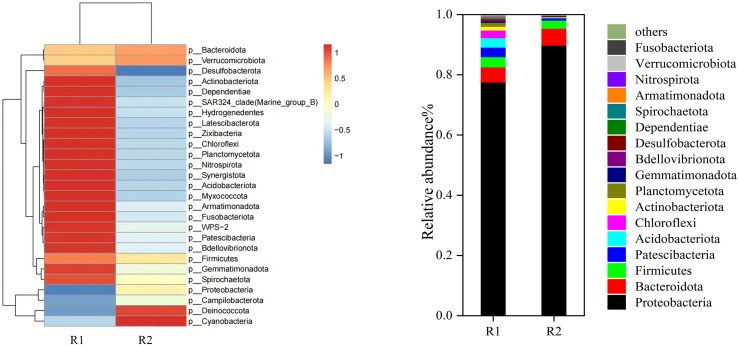
Bacterial composition and abundance at the phylum level.

The diversity and abundance of microbial communities on particle electrodes in different reactors were analyzed using 16S rRNA gene high-throughput sequencing. Fig. 10 presented the specific composition of microbial communities at each taxonomic level, along with microbial diversity indices. The microbial richness index was significantly lower in the 3D-BER. The dominant phylum in R1 were Proteobacteria, Bacteroidota, Firmicutes, Patescibacteria, and Acidobacteria, with relative abundances of 77.52%, 5.32%, 3.38%, 3.15%, and 3.16%, respectively. The dominant phylum in R2 were Proteobacteria, Bacteroidota, and Firmicutes, with relative abundances of 89.79%, 5.61%, and 2.69%, respectively. Many common denitrifying bacteria have been reported in Proteobacteria, such as *Thauera*, *Hydrogenophaga*, *Alcaligenes*, and *Dechloromonas*.^31^ Bacteroidota was a common nitrate-reducing bacteria phylum that could utilize a variety of carbon sources to degrade complex organic matter. Chloroflexi encompassed a group of ecologically and physiologically varied bacteria that have been found in many anaerobic settings.^32^ Firmicutes has also been shown to be associated with hydrogen autotrophic denitrification and could denitrify in environments with little organic matter.^33,34^ Under electrical stimulation, the relative abundance of Acidobacteriota decreased (R1: 3.16%%, R2: 0.06%). Some genera in Acidobacteriota utilized organic and inorganic nitrogen sources under anaerobic conditions, and there was evidence for an ecological connection between Acidobacteriota and Proteobacteria.^35^ Ward investigated the denitrification effects of three different strains of acidogenic bacteria and found that all three genomes showed a reduction in nitrate (Fig. 11).^36^

**Fig. 12:**
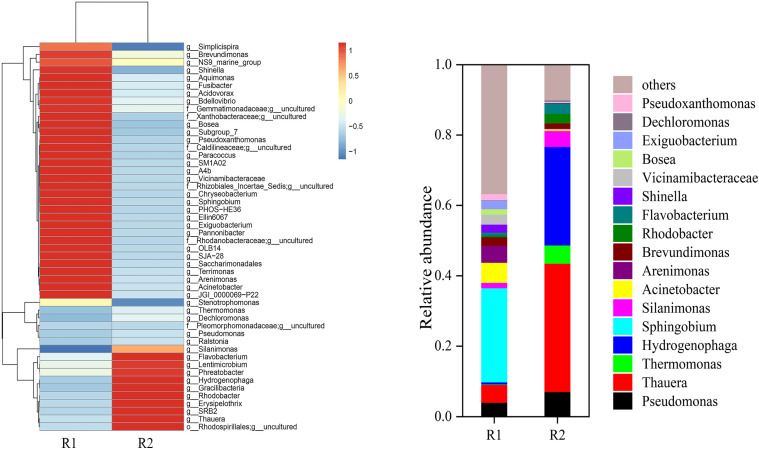
Composition and abundance of the bacteria at genus level.

The dominant genera in R1 were *Sphingobium*, *Thauera*, *Acinetobacter*, *Arenimonas*, and *Vicinamibacteraceae*, with relative abundances of 26.73%, 5.07%, 5.69%, 4.48%, and 2.089%, respectively. *Arenimonas* and *Vicinamibacteraceae* all used organic carbon sources for heterotrophic denitrification. The dominant genera in R2 were *Thauera*, *Hydrogenophaga*, *Acinetobacter*, and *Pseudomonas*, with relative abundances of 36.39%, 27.99%, 5.64%, and 7.09%, respectively. *Sphingobium* dropped dramatically from 26.73% in R1 to 0.02% in R2, which was probably why this genus cannot tolerate the applied current. The enrichment of *Thauera* (R1: 5.07%, R2: 36.39%) under electrified conditions also indicated that electrical stimulation promoted the enrichment of dominant denitrifying bacteria. Yang found that in the complex oxygen environment of the SCMFC electrode biofilm, *Thauera* could be highly enriched and played an essential role in denitrification and electron transfer.^37^*Acinetobacter* (R1: 5.69%, R2: 5.64%) could perform denitrification under anaerobic conditions, and Su's research found that N_2_O was not detected spiked with Acinetobacter, suggesting that NO_3_^−^–N might be completely converted to N_2_ in the reactor.^38^*Pseudomona*s was a well-known heterotrophic denitrifying bacterium that could produce oscillatory denitrification by co-respiring NO_3_^−^–N and NO_2_^−^–N with regulated O_2_ supplies.^39^*Rhodococcus* (R1: 0.49%, R2: 2.71%) could grow autotrophically and reduce nitrate under anaerobic conditions. In addition, *Rhodococcus* was capable of mineral bioleaching and had a broad catabolic diversity. The relative abundance of *Thermomonas* increased from 0.10% in R1 to 5.19% in R2. It was same with previous experimental results in which Thermomonas grew rapidly and played an essential role in the CEAD reactor.^40^*Hydrogenophaga* (R1: 0.59%, R2: 27.99%) could use hydrogen as an electron donor for autotrophic denitrification, and CO_2_ produced by heterotrophic denitrifying bacteria could promote the growth of *Hydrogenophaga* in the 3D-BER (Fig. 12).

**Fig. 13:**
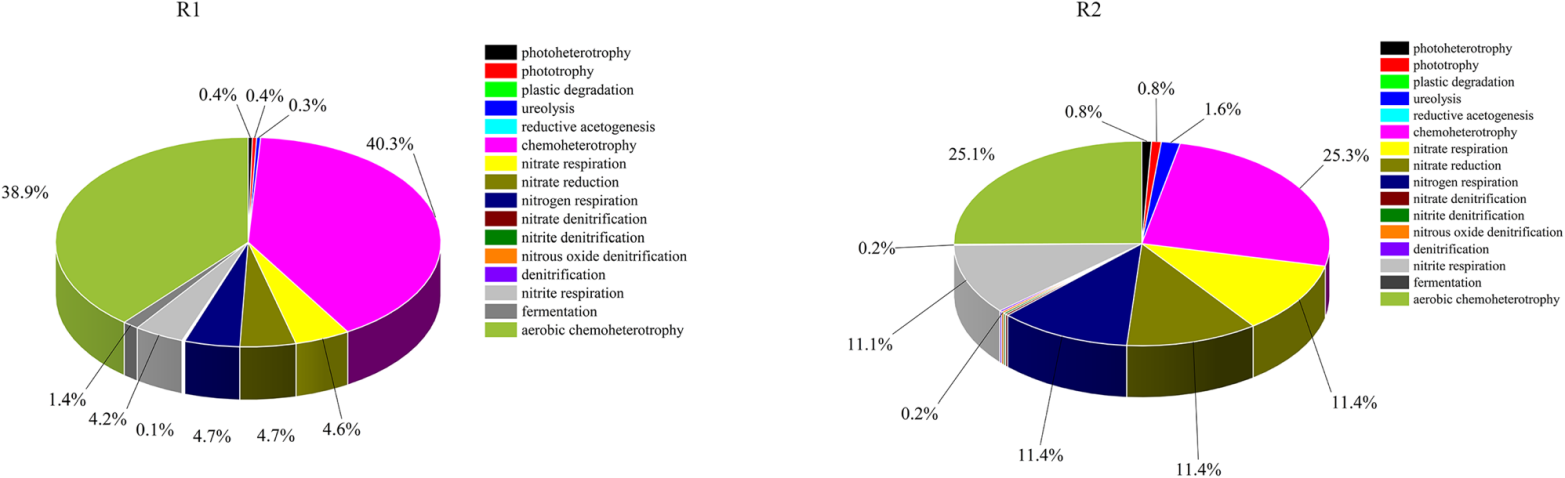
Functional annotation of prokaryotic taxa (FAPROTAX).

In this experiment, the prediction of gene function was performed using functional annotation of prokaryotic taxa (FAPROTAX), a database that connects metabolic or ecologically important activities to different taxa of bacteria or archaea, based on research on culture representation.^41^ As shown in Fig. 13, the relative abundance of chemical heterotrophic microorganisms in R1 was significantly higher (40.3%) than that in R2 (25.3%). The relative abundances of nitrate-reducing bacteria, nitrite-reducing bacteria, and nitrate-denitrifying bacteria in R2 were higher than those in R1. Appropriate electrical stimulation promoted the enrichment of dominant bacteria, such as hydrogen autotrophic denitrification, heterotrophic denitrification, and electroactive bacteria, thereby reducing the diversity and uniformity of the microbial community. The energization conditions changed the species and abundance of the main autotrophic denitrifying bacteria in the two reactors. The distribution of bacteria was significantly different in species and relative abundance. In addition, heterotrophic denitrifying bacteria generally exhibited faster growth rates than autotrophic denitrifying bacteria. However, in conditions where the COD/N ratio was low, the contribution of autotrophic denitrifying bacteria became more pronounced, thereby limiting the enrichment of heterotrophic denitrifying organisms.

## Conclusion

This study presented a promising technique for enhancing denitrification performance with a limited organic carbon source. In a 3D-BER that combined heterotrophic and autotrophic denitrification, the nitrate removal rate exceeded 80% at the ranges of *I* (30–60 mA), HRT (8–12 h), and COD/N (1–2.5). When the COD/N = 1–2.5, both autotrophic and heterotrophic denitrifying bacteria played essential roles, heterotrophic denitrification removed most of nitrate at the COD/N > 2.5. It was suggested that the optimal values for HRT and electric current were 8 h and 40 mA, respectively. The electric current stimulated the growth of denitrifying bacteria. On the particle electrode, more denitrifying bacteria were enriched, particularly *Thauera*, *Pseudomonas*, and *Hydrogenophaga*, which were crucial to the denitrification process.

## Conflicts of interest

There are no conflicts to declare.

## Supplementary Material

RA-013-D3RA01403G-s001
