# Supplementary material for: Study of a three-dimensional biofilm-electrode reactor (3D-BER) that combined heterotrophic and autotrophic denitrification (HAD) to remove nitrate from water
Source: RSC Adv. 2023 May 15;13(21):14675–84. doi: 10.1039/d3ra01403g (PMC10183716; doi:10.1039/d3ra01403g)

Electronic Supplementary Material (ESI) for RSC Advances.
This journal is © The Royal Society of Chemistry 2023

Supplementary Materials

1 **Method for Measuring NO_3^--N**

Preparation of the adsorption column: The new large-pore neutral resin is first washed twice with 200 ml of water, soaked in methanol overnight, the methanol is discarded, and then washed twice with 40 ml of methanol. The resin is then washed with fresh deionized water until the liquid flowing out of the column into a beaker is no longer milky white. When packing the resin into the column, no air bubbles should be present between the resin particles.

Take 200 ml of water sample and place it in a conical flask or beaker, add 2 ml of zinc sulfate solution, and add sodium hydroxide solution dropwise under stirring to adjust the pH to 7. Alternatively, adjust the pH of a 200 ml water sample to 7, and then add 4 ml of aluminum hydroxide suspension. After the flocculated gel has settled or has been centrifuged, take 100 ml of the supernatant and wash the adsorption resin column twice, with a flow rate of 1 to 2 drops per second, maintaining consistent flow rates between samples, and discard. Continue to pass the water sample supernatant through the column and collect 50 ml in a colorimetric tube for determination. The resin is washed three times with 150 ml of water and reserved for use. The adsorption capacity of the resin is large and can handle 50 to 100 surface water samples, depending on the organic matter content. After multiple uses, the resin can be regenerated with methanol if the absorbance at 220 nm and 275 nm wavelengths, measured using fresh deionized water that has not been in contact with rubber products, is close to zero and exceeds the instrument's allowable error.

Add 1.0 ml of hydrochloric acid solution and 0.1 ml of amino sulfonic acid solution to the colorimetric tube. When the nitrite nitrogen concentration is below 0.1 mg/L, the addition of amino sulfonic acid solution can be omitted. Using a 10 mm quartz cuvette, measure the absorbance at 220 nm and 275 nm wavelengths, with 50 ml of resin-adsorbed fresh deionized water and 1 ml of hydrochloric acid solution as a reference. Calibration curve construction: add 0.50, 1.00, 2.00, 3.00, and 4.00 ml of nitrate nitrogen standard stock solution to five 200 ml volumetric flasks, respectively,

and dilute to the mark with fresh deionized water. The mass concentrations are 0.25, 0.50, 1.00, 1.50, and 2.00 mg/L nitrate nitrogen, respectively. Measure the absorbance following the same procedure as for water sample determination.

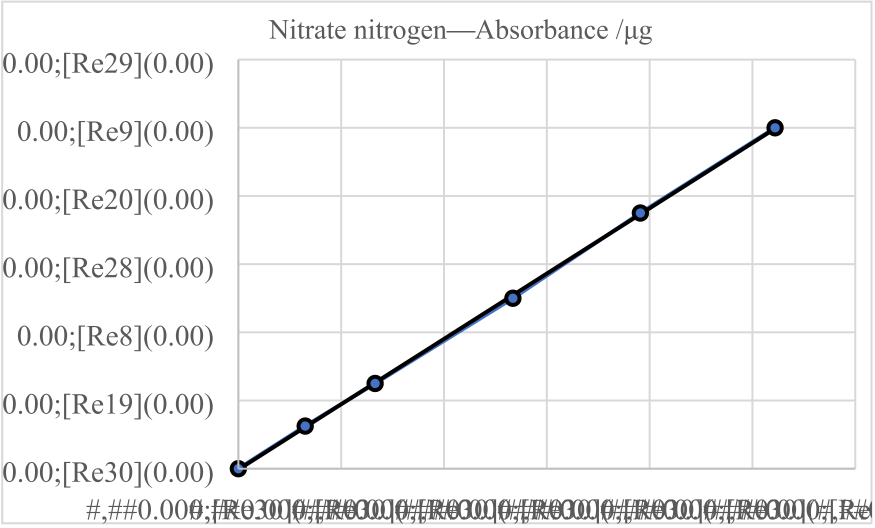

2 **Method for Measuring NO_2^--N**

In water samples, nitrite nitrogen (NO_2^--N) undergoes diazotization with sulfanilamide, and then couples with N-(1-naphthyl) ethylenediamine dihydrochloride (NED) to form a water-soluble purple-red azo compound. The absorbance of the peak at a wavelength of 543 nm can be measured, which allows for the quantification of nitrite nitrogen in water samples.

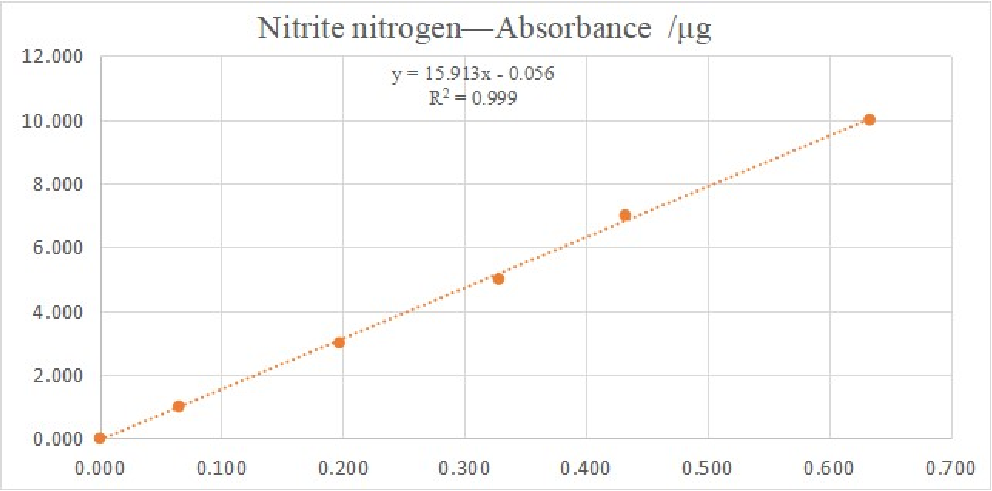

3 **Method for Measuring TN**

The principle of determining total nitrogen using the alkaline potassium persulfate digestion method is that, in a certain temperature aqueous solution, potassium persulfate undergoes a reaction to produce potassium bisulfate and atomic oxygen. Potassium bisulfate dissociates in the solution to produce hydrogen ions, thus the decomposition process can be accelerated to completion in the alkaline medium of sodium hydroxide . The atomic oxygen released under the conditions of 120℃ to 124℃ can convert nitrogen elements in nitrogen-containing compounds in the water sample into nitrate. During this process, organic matter is also oxidized and decomposed. According to GB636-2012 alkaline potassium persulfate digestion-ultraviolet spectrophotometry method, prepare potassium nitrate standard solution (10 mg/L), take 0, 0.2, 0.5, 1, 3, and 7 ml of potassium nitrate standard solution and add ammonia-free water to 10 ml. Then add 5 ml of alkaline potassium persulfate solution, place in a sterilizer, and digest at 120℃ for 30 minutes. After digestion, remove, cool, and add 1 ml of (1:9) hydrochloric acid solution, dilute to 25 ml with ammonia-free water, shake well, and measure the absorbance at wavelengths 220 nm and 275 nm using 10 mm cuvettes. Absorbance = $A_{220} - 2A_{275}$.

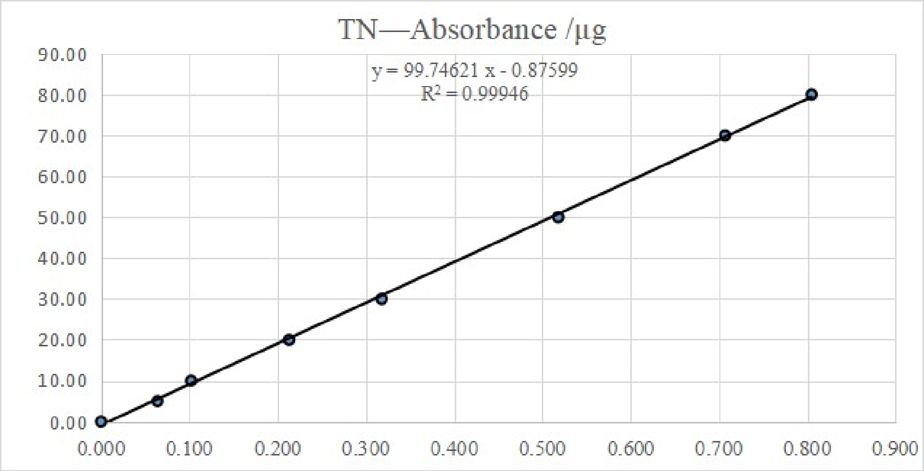

Supplement: RA-013-D3RA01403G-s001 [file RA-013-D3RA01403G-s001.pdf]
